# The chemokine CXCL16 modulates neurotransmitter release in hippocampal CA1 area

**DOI:** 10.1038/srep34633

**Published:** 2016-10-10

**Authors:** Maria Amalia Di Castro, Flavia Trettel, Giampaolo Milior, Laura Maggi, Davide Ragozzino, Cristina Limatola

**Affiliations:** 1 Istituto Pasteur-Italia, Dipartimento di Fisiologia e Farmacologia, Sapienza Università di Roma, Piazzale Aldo Moro 5, 00185 Roma, Italy; 2 Dipartimento di Fisiologia e Farmacologia, Sapienza Università di Roma, Piazzale Aldo Moro 5, 00185 Roma, Italy; 3 Neuromed I.R.C.C.S., Via Atinese 18, 86077 Pozzilli (IS), Italy

## Abstract

Chemokines have several physio-pathological roles in the brain. Among them, the modulation of synaptic contacts and neurotransmission recently emerged as crucial activities during brain development, in adulthood, upon neuroinflammation and neurodegenerative diseases. CXCL16 is a chemokine normally expressed in the brain, where it exerts neuroprotective activity against glutamate-induced damages through cross communication with astrocytes and the involvement of the adenosine receptor type 3 (A3R) and the chemokine CCL2. Here we demonstrated for the first time that CXCL16 exerts a modulatory activity on inhibitory and excitatory synaptic transmission in CA1 area. We found that CXCL16 increases the frequency of the miniature inhibitory synaptic currents (mIPSCs) and the paired-pulse ratio (PPR) of evoked IPSCs (eIPSCs), suggesting a presynaptic modulation of the probability of GABA release. In addition, CXCL16 increases the frequency of the miniature excitatory synaptic currents (mEPSCs) and reduces the PPR of evoked excitatory transmission, indicating that the chemokine also modulates and enhances the release of glutamate. These effects were not present in the A3RKO mice and in WT slices treated with minocycline, confirming the involvement of A3 receptors and introducing microglial cells as key mediators of the modulatory activity of CXCL16 on neurons.

Chemokines are a large group of proteins originally identified for their chemotactic and regulatory activities in the immune system. More recently, chemokines and their receptors were identified in the nervous system as key mediators of homeostatic brain functions such as developmental processes^1^,^2^,^3^, neurotransmission^4^,^5^,^6^,^7^, in addition to neuroinflammation^8^,^9^,^10^,^11^,^12^,^13^, neurodegeneration^14^,^15^,^16^,^17^ and cancer^18^,^19^. The chemokine CXCL16 was originally discovered as a scavenger receptor for oxidized lipoprotein (therefore termed SR-PSOX22) and, independently, as ligand for the CXC-chemokine receptor CXCR6/Bonzo (also termed TYMSTR, STRL33)^20^,^21^. It is synthesized as a transmembrane multi-domain molecule consisting of a chemokine module fused to a glycosylated mucin-like stalk and a single transmembrane helix. A soluble version of CXCL16 is generated by constitutive or inducible cleavage of the transmembrane form through the action of cell-surface proteases ADAM10 and 17 (ADAM, a disintegrin and metalloproteinase)^22^,^23^,^24^.

CXCL16 is highly expressed in the brain during pathological conditions like multiple sclerosis, glioma, schwannomas and meningiomas^23^,^25^,^26^,^27^,^28^,^29^,^30^; moreover, CXCL16/CXCR6 signaling has been recently described to play a crucial role in counteracting brain glutamate excitotoxic damage upon cerebral ischemia. The mechanisms underlying the neuroprotective activity of CXCL16 require the interplay between microglia, astrocyte and neurons and the activity of the Adenosine receptor type 3 (A3R), with consequent release of CCL2 by glial cells^31^,^32^. Besides being upregulated in phatological conditions, CXCL16 and its unique receptor CXCR6 are physiologically expressed by cells of the brain parenchima, such as astrocytes, microglia and neurons^31^,^32^ suggesting a possible role played by this chemokine in brain homeostasis. To investigate the physiological role of CXCL16/CXCR6 axis we examined the ability of CXCL16 to modulate neurotransmission. We focused on the inhibitory and excitatory synaptic currents recorded from CA1pyramidal neurons in acute hippocampal slices by whole-cell patch clamp techniques. We found that CXCL16 is able to modulate neurotransmitter release on both GABA-ergic and glutamatergic synapses with a mechanism that requires functional A3R, and the contribution of microglia.

## Results

### CXCL16 enhances spontaneous GABA release onto hippocampal CA1 pyramidal cells

**Figure 1:**
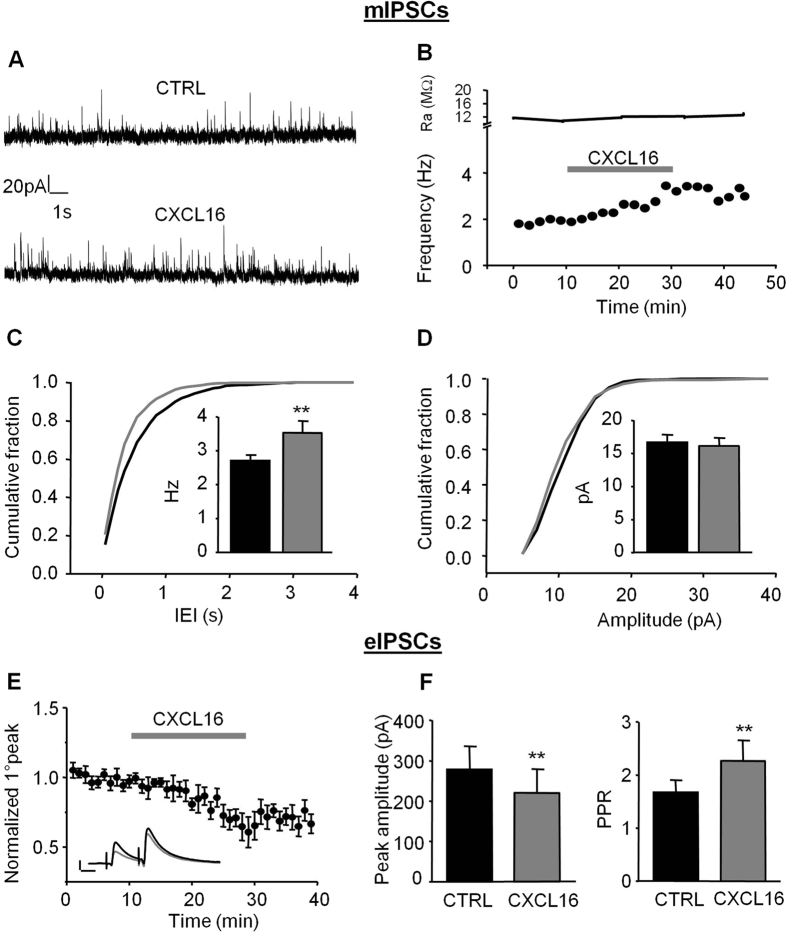
Bath application of CXCL16 alters inhibitory synaptic transmission in CA1 pyramidal neurons. (**A**) Representative traces of mIPSCs recordings in control condition and in presence of CXCL16 (holding current 0 mV). We observed an increase in the frequency of the events without changes in the amplitude. (**B**) Same cell as in (**A**) time course of the effect of CXCL16 on the mIPSCs frequency (lower graph) and series resistance (upper trace). (**C**) Cumulative probability histogram for inter-mIPSC interval (IEI) during baseline (black) and CXCL16 (grey) conditions. Inset: mean frequency from 8 cells (*p < 0.05, Student’s paired *t* test). (**D**) Cumulative probability histogram for mIPSC amplitude during baseline (black) and CXCL16 treatment (grey). No changes in the mean mIPSC amplitude histogram (inset). (**E**) Mean effect of CXCL16 on amplitude of first GABA-IPSC for 10 cells. Error bars indicate SEM. CXCL16 (10 nM) was applied for 20 min. Inset: Representative traces showing that bath application of CXCL16 (grey line) decreases the amplitude of the first GABA-IPSCs in paired pulse experiments. Each trace in this and other figures is the mean of the last 10 sweeps in each condition. Horizontal bar: 25 ms, vertical bar: 100 pA. (**F**) Means of the amplitude of the first GABA-IPSC and of the paired-pulse ratio (PPR) in each condition (Student’s paired *t* test, *p < 0.05; **p < 0.01).

To investigate the role of CXCL16 on GABAergic transmission, we recorded miniature GABAergic currents from pyramidal neurons in the CA1 area. As shown in Fig. 1A,B, after about 10 min of application, CXCL16 starts to increase the frequency of mIPSCs. This effect was long-lasting, with a slow wash, and is similar to what was observed for other chemokines affecting synaptic transmission^33^,^34^,^35^,^36^. The modulation of mIPSCs frequency was also evident as a leftward shift of the cumulative probability plot for the inter-event intervals (IEI) and as a significant increase in the mean mIPSC frequency (CTRL: 2.71 ± 0.17 Hz, CXCL16: 3.52 ± 0.36 Hz, N = 8, p = 0.008, Fig. 1C). In contrast, CXCL16 did not alter mIPSC amplitude (CTRL: 16.67 ± 1.19 pA, CXCL16: 16.18 ± 1.21 pA, N = 8, p = 0.29, Fig. 1D). These data indicate that CXCL16 enhances the spontaneous GABA release at inhibitory synapses impinging onto CA1 pyramidal cells. As a control for specificity, bath application of CXCL16 in CXCR6 KO slices, did not affect the frequency (CTRL: 4.55 ± 0.31 Hz, CXCL16 4.73 ± 0.22 Hz, N = 4, p = 0.37) and the amplitude (CTRL 17.55 ± 2.19 pA, CXCL16 16.62 ± 1.83 pA, N = 4, p = 0.23) of miniature IPSCs, contrary to what observed in sibling controls where CXCL16 increased mIPSCs frequency (CTRL: 2.97 ± 0.26 Hz, CXCL16 4.05 ± 0.53 Hz, N = 4. p = 0.04, N = 4) without altering amplitude (CTRL: 17.91 ± 1.11; CXCL16: 18.34 ± 0.84; p = 0.29; N = 4).

### CXCL16 regulates evoked GABA release

We then investigated whether CXCL16 regulates GABA responses evoked by extracellular stimulation with paired stimuli (inter stimulus interval = 50 ms) in the stratum radiatum of CA1 hippocampal region. Bath application of CXCL16 (10 nM) reduced the amplitude of the evoked IPSC (eIPSC) to 72.7 ± 8% of control within 20 min (Fig. 1E,F left panel, CTRL: 277.78 ± 57.85 pA, CXCL16: 219.79 ± 59.27 pA, N = 10, p = 0.003) and significantly increased the paired pulse ratio (PPR) from 1.67 ± 0.30 to 2.43 ± 0.48 (p = 0.03, N = 10; Fig. 1F, right panel) suggesting that CXCL16 acts presynaptically to reduce the evoked GABA release. Similar experiments were repeated on homozygous CXCR6 knock-out mice where CXCL16 failed to affect the paired pulse ratio (CTRL: 1.29 ± 0.23 pA, CXCL16: 1.19 ± 0.13 pA, N = 4, p = 0.37) and the amplitude of eIPSCs (CTRL: 195.18 ± 73.63 pA, CXCL16: 177.25 ± 66.36 pA, N = 4, p = 0.101). In littermate controls the chemokine affected both PPR (CTRL: 1.24 ± 0.24 pA, CXCL16: 2.39 ± 0.14 pA, N = 3, p = 0.017) and amplitude of eIPSCs (CTRL: 199.2 ± 44.37 pA, CXCL16: 100.48 ± 30.96 pA, N = 3, p = 0.021) as in WT animals.

**Figure 2:**
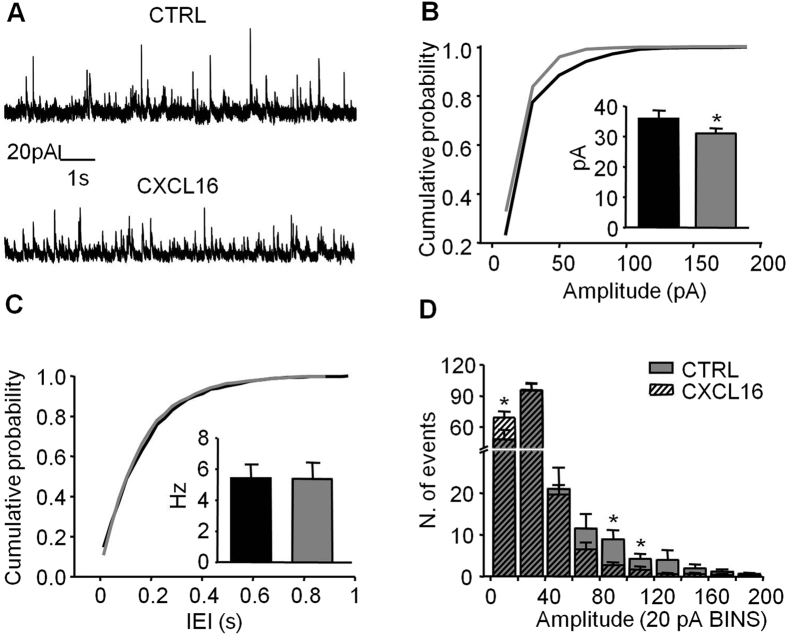
Bath application of CXCL16 decreases the amplitude of spontaneous IPSCs. (**A**) Representative traces of spontaneous IPSCs recordings from a cell in control and CXCL16 condition. (**B**) Same cell as in (**A**) cumulative probability histogram for amplitude of sIPSCs in control (black) and CXCL16 (grey). Shift toward the left indicates a decrease of the amplitude in CXCL16 condition. Inset. Mean of the amplitude in both conditions (N = 7, Student’s paired *t* test: p < 0.05). (**C**) Cumulative probability histogram for IEI of sIPSCs in control (black) and CXCL16 (grey). No effect of CXCL16. Inset. Means of IEI. (**D**) Average amplitude distribution showing that after CXCL16 treatment the number of small sIPSCs (<20 pA) is increased whereas larger ones (>80 pA) is decreased. Bin width is 20 pA. *p < 0.05.

The results obtained on the evoked release of GABA are apparently different from those on miniature recordings, as CXCL16 application increased the spontaneous GABA release events. To better address this issue, we investigated the effect of CXCL16 application on spontaneous GABA currents (sIPSCs). Bath application of CXCL16 decreased the mean sIPSCs amplitude (CTRL: 35.85 ± 2.76 pA, CXCL16: 31.01 ± 1.69 pA, N = 7, p = 0.039) and shifted toward the left the cumulative distribution histogram (Fig. 2A,B). Although the mean frequency of sIPSCs did not change upon CXCL16 application (CTRL: 5.43 ± 0.89 Hz, CXCL16: 5.37 ± 1.05 Hz, N = 7, p = 0.88, Fig. 2C), closer inspection of the traces revealed that the number of large amplitude sIPSCs is reduced, whereas the number of small-amplitude sIPSCs is increased. This effect was quantified in a pooled amplitude histograms showing that the number of events falling within 20 pA (the average of mIPCs amplitude) was increased in the presence of CXCL16 whereas the number of events larger than 80 pA was decreased (Fig. 2D). Overall, these results support the evidence that CXCL16 is able (i) to produce a presynaptic increase of the probability of spontaneous, action potential-independent GABA release, because the frequency of both the smallest sIPSCs and mIPSCs is increased and (ii) to reduce action-potential dependent GABA release, as demonstrated by the decrease in the amplitude of both eIPSCs and large sIPSCs.

In order to elucidate possible mechanisms underlying the effects of CXCL16 on evoked GABAergic activity, we investigated the role of metabotropic GABA-B receptor that has been reported to specifically reduce the release of evoked GABA onto CA1 pyramidal neurons^37^,^38^,^39^.

**Figure 3:**
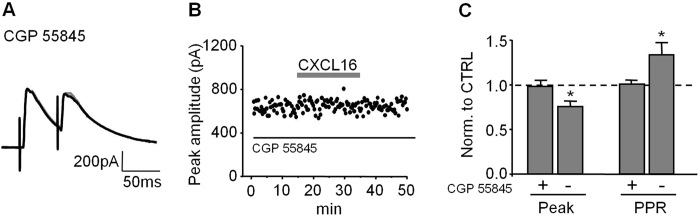
The GABA-B antagonist CGP 55845 blocks the CXCL16-dependent modulation of evoked GABAergic transmission. (**A**) Representative traces of evoked GABA currents in paired pulse experiments performed in presence of CGP 55845 (1 μM) are similar in CTRL (black line) and CXCL16 (grey line) condition. Each trace is the mean of the last 10 sweeps in each condition (**B**) Time course of the first GABA-IPSC amplitude. Bath application of CXCL16 10 nM for 20 min does not produces effect when slices were pre-treated with CGP 55845. (**C**) Means of the peak amplitude and paired-pulse ratio (PPR) after CXCL16 treatment, in presence and in absence of CGP 55845. Values are expressed as fold increase relative to CTRL. (Student’s paired *t* test, **p < 0.01).

When the slices were pre-incubated for 30 min with the GABA-B receptors antagonist CGP 55845 (1 μM) and then continuously superfused, CXCL16 was not able to affect the peak amplitude and the PPR of eIPSCs (fold increases relative to baseline: peak amplitude: 0.98 ± 0.07, p = 0.73; PPR: 1.01 ± 0.04, p = 0.41, N = 6, Fig. 3). By contrast, in parallel experiments were CGP55845 was not applied, CXCL16 reduced the peak amplitude to 0.75 ± 0.06 (fold increase relative to CTRL, N = 6, p = 0.02) and increased the eIPSC PPR to 1.33 ± 0.14 (N = 6, p = 0.03). Together these results suggest that the inhibitory effect of CXCL16 on evoked GABAergic transmission is mediated by the GABA-B receptors activity.

### Excitatory pre-synaptic transmission is potentiated by CXCL16 application

The results presented above showed that CXCL16 affects inhibitory synaptic transmission by modulating GABA release. To further investigate its action on synaptic transmission, we tested the effects of CXCL16 on glutamatergic one.

**Figure 4:**
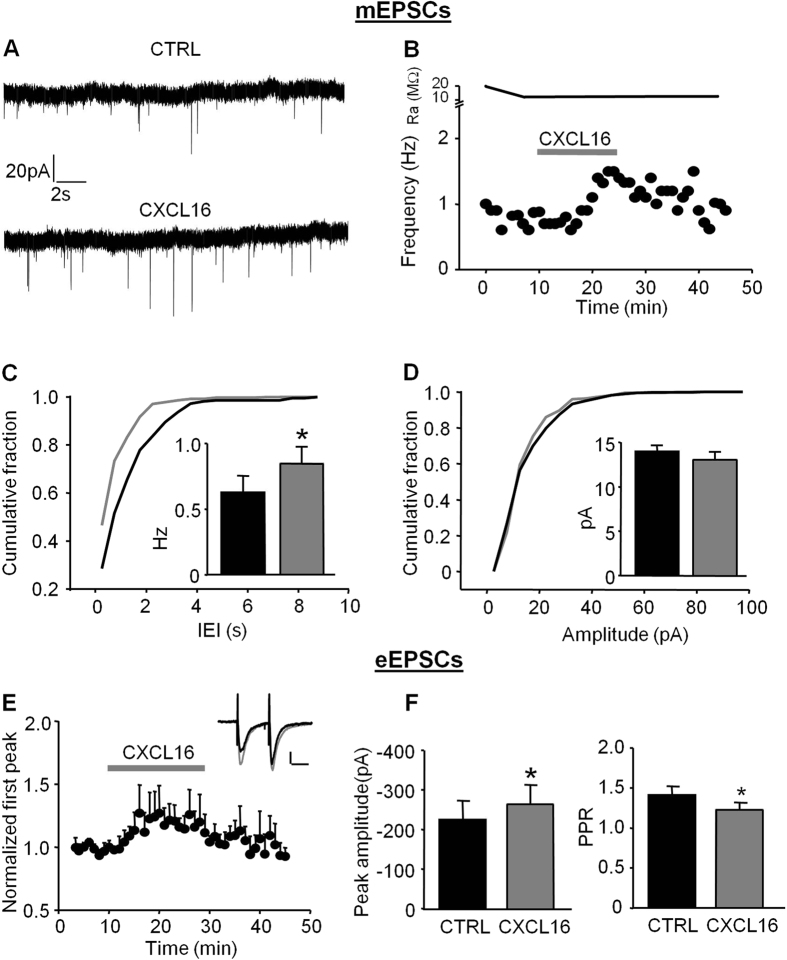
CXCL16 positively modulates excitatory synaptic transmission in CA1 pyramidal neurons. (**A**) Representative traces of miniature excitatory synaptic current (mEPCs) from a cell before and after CXCL16 treatment. (**B**) Same cell as in (**A**) time course analysis showing that CXCL16 increases the mEPSCs frequency during 20 min of application. In the upper trace Series resistance. (**C**) Cumulative probability plot for mEPSCs amplitude and histograms of the means (inset) showing no effect after CXCL16 treatment (grey line, N = 8). (**D**) Cumulative probability plot for IEI is shifted towards the left in presence of CXCL16 (grey), indicating a presynaptic effect of this chemokine. The average frequency of mEPSCs is significantly increased after CXCL16 treatment (N = 8, Student’s *t* test, *p < 0.05) (**E**) Time course of the effect of CXCL16 on first AMPA-EPSCs evoked by extracellular stimulation. Error bars indicate SEM. CXCL16 (10 nM) was applied for 20 min. Inset: Representative traces showing that bath application of CXCL16 (grey line) increases the amplitude of first peak in paired pulse experiments. Horizontal bar: 25 ms, vertical bar: 25 pA. (**F**) Means of the amplitude of the first AMPA-EPSCs in each condition and means of the PPR (N = 7, Student’s paired *t* test, *p < 0.05; **p < 0.01).

CXCL16 increased the frequency of miniature excitatory post-synaptic currents (mEPSCs), as illustrated in the time course from a representative experiment showed in Fig. 4A,B. Specifically, during bath application of CXCL16 (10 nM, 20 min) the cumulative distribution curve for IEIs was shifted towards the left and, correspondingly, the average frequency of mEPSC was increased (CTRL: 0.62 ± 0.13 Hz, CXCL16: 0.85 ± 0.13 Hz, N = 8, p = 0.05, Fig. 4C and inset). In contrast the mean amplitudes of mEPSCs were similar before and after CXCL16 treatment (15.03 ± 0.58 pA and 14.26 ± 1.14 pA, respectively, N = 8, p = 0.2, Fig. 4D inset) and no changes in the cumulative probability plot for amplitudes were observed (Fig. 4D). These results indicate that CXCL16 is able to increase the spontaneous glutamate release onto CA1 pyramidal neurons.

In addition, we elicited excitatory post-synaptic currents by Schaffer collateral stimulation (eEPSCs) with paired stimuli (ISI = 50 ms). We found that CXCL16 significantly increased the peak amplitude of eEPSC (CTRL: 224.69 ± 48.45 pA, CXCL16: 258.24 ± 47.88 pA, N = 7, p = 0.025, Fig. 4E,F left panel) and reduced the PPR (CTRL: 1.41 ± 0.11, CXCL16: 1.26 ± 0.08, N = 7, p = 0.025, Fig. 4F right panel), implying that CXCL16 acts increasing the probability of evoked glutamate release.

### CXCL16 fails to affect neurotransmission in adenosine receptor A3R Knock-Out mice

**Figure 5:**
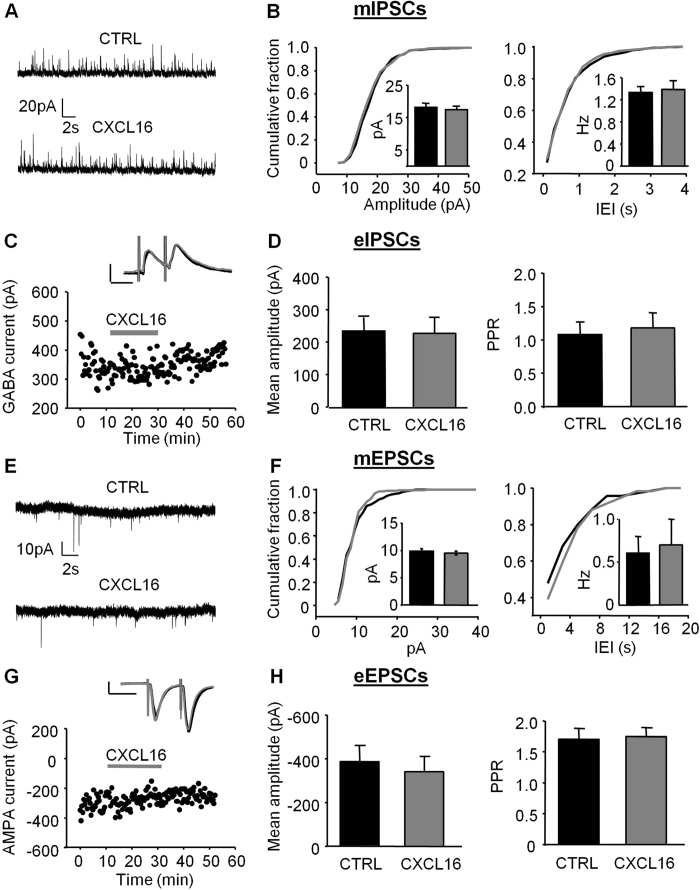
Bath application of CXCL16 is ineffective on inhibitory and excitatory synaptic transmission in A3RKO mice. (**A**) Representative traces of a mIPSC recording from a pyramidal CA1 neuron in slices prepared from A3RKO mice. (**B**) Cumulative distribution histograms for mIPSCs amplitude and IEI showing no effect of CXCL16 (grey line). In the insets mean amplitude and mean frequency (N = 5, Student’s *t* test, p > 0.05) in control (black) and CXCL16 (grey). (**C**) Example of a time course of first GABA-IPSC amplitude in a paired pulse experiment. Bath application of CXCL16 10 nM for 20 min does not produces any effect. Example traces are average of 10 consecutive sweeps for each condition. Black line: CTRL, grey line: CXCL16. Horizontal bar: 100 pA, vertical bar: 40 ms (**D**) Means of the first GABA-IPSC and paired pulse ratio showing no changes after CXCL16 treatment (N = 5). (**E**) Representative traces of a mEPSC recording from a pyramidal CA1 neuron in slices prepared from A3RKO mice. (**F**) Cumulative distribution curves for mEPSCs amplitudes and IEI are similar before (black line) and after CXCL16 treatment (grey line). Inset: histograms of the means for mEPSCs amplitude and frequency. (**G**) Time course of eEPSC showing no effect of CXCL16 application. Inset: example traces as in C. (**H**) means of the first AMPA-EPSC and paired pulse ratio (N = 5).

We previously reported that CXCL16 exerts neuroprotective effects through the activation of the A3R on astrocytes^31^. Here we examined the effect of the chemokine on synaptic transmission in slices obtained from A3R KO mice. As shown in Fig. 5A,B, in A3RKO mice, CXCL16 failed to produce any change in the cumulative distribution curves for mIPSCs amplitude and IEIs and did not modify the mean amplitude (CTRL 18.17 ± 1.21 pA; CXCL16 17.41 ± 1.08, N = 5, p = 0.41, N = 5) or the frequency (CTRL: 1.33 ± 0.11, CXCL16: 1.39 ± 0.15, N = 5, p = 0.54) of mIPSCs (Fig. 5A,B). In addition, CXCL16 did not alter the amplitude of eIPSCs (CTRL: 233.28 ± 42.37 pA; CXCL16 227.08 ± 46.96 pA, N = 5, p = 0.62) and the PPR (CTRL: 1.08 ± 0.19, CXCL16 1.18 ± 0.23, N = 5, p = 0.08) (Fig. 5C,D).

Recordings of miniature glutamatergic currents showed that CXCL16 was unable to modulate mEPSCs amplitude (CTRL: 10.58 ± 0.62 pA, CXCL16 10.29 ± 0.85 pA, N = 7 p = 0.65) or frequency (CTRL: 0.52 ± 0.15, CXCL16: 0.67 ± 0.27, N = 7, p = 0.47) in A3R KO slices (Fig. 5E,F inset). In addition no differences in the cumulative probability plot for amplitude and IEI were detected after CXCL16 application (Fig. 5F).

Similarly, in A3RKO slices the amplitude and PPR of eEPSCs were unchanged by chemokine application (CTRL: 371.01 ± 59.82 pA, CXCL16: 342.6 ± 70.33 pA, p = 0.17, N = 5; CTRL 1.71 ± 0.18 CXCL16 1.75 ± 0.15, p = 0.61, N = 5, Fig. 5G,H).

**Figure 6:**
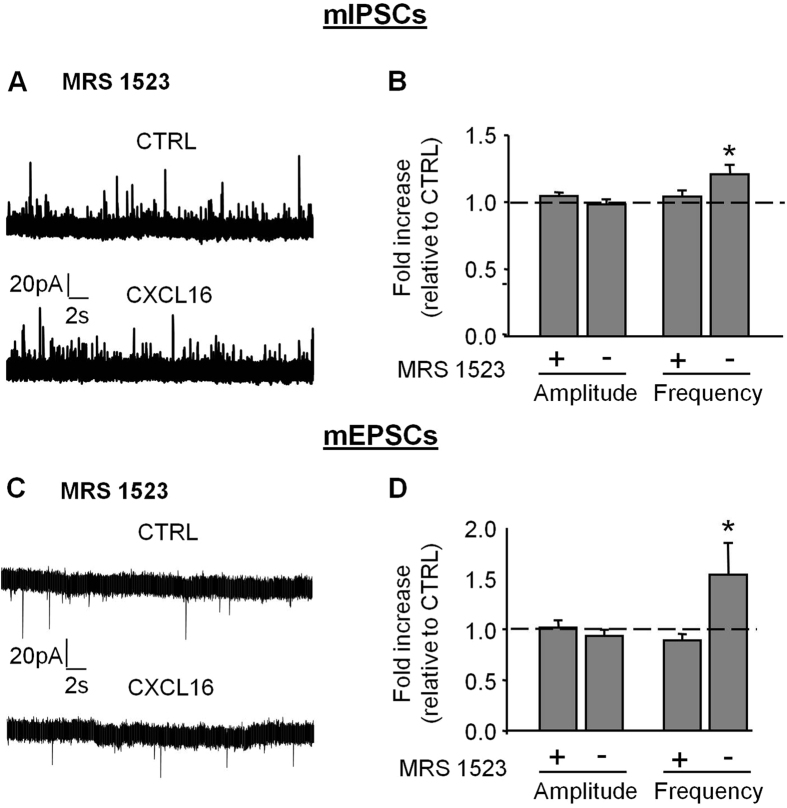
The selective A3R antagonist MRS 1523 prevents CXCL16-dependent modulation of synaptic transmission. (**A**) Representative traces of miniature GABAergic currents recorded in presence of MRS 1523 (100 nM), before and during CXCL16 treatment. (**B**) Relative means for mIPSCs amplitude and frequency (N = 6) during CXCL16 application in presence and absence of MRS 1523. Values are expressed as fold increase compared to CTRL. No changes for peak amplitude and PPR when CXCL16 was applied in presence of MRS 1523. (**C**) Representative recordings of mEPSCs in the same condition as in (**A**). (**D**) Histograms for mEPSCs amplitude and frequency (N = 6), CTRL: black, CXCL16: grey. (Student’s paired t test,*p < 0.05).

To further investigate the involvement of A3R, brain slices obtained from wt mice were treated with the selective A3R antagonist MRS1523 (100 nM, 30 min pre-treatment, N = 6, Fig. 6), and stimulated with CXCL16. In this condition, CXCL16 did not affect either the amplitude or the frequency of mIPSCs (fold increases relative to CTRL: amplitude: 1.05 ± 0.03, p = 0.14; frequency: 1.04 ± 0.05, p = 0.39, N = 6, Fig. 6A,B) and mEPSCs (fold increases relative to CTRL: amplitude: 1.02 ± 0.07, p = 0.81; frequency: 0.89 ± 0.07, p = 0.09, N = 6, Fig. 6C,D), confirming that A3R activity is involved with CXCL16 effects on basal neurotransmission.

### Minocycline treatment prevents CXCL16-dependent modulation of synaptic transmission

**Figure 7:**
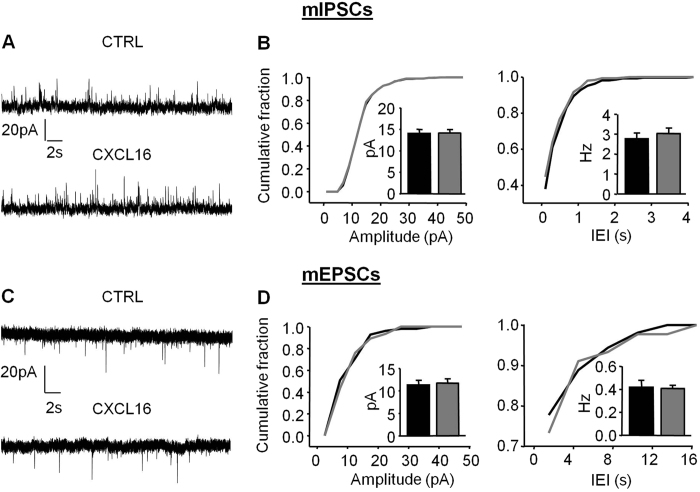
Minocycline treatment blocks CXCL16-dependent effects on basal synaptic transmission. (**A**) Representative traces of miniature GABAergic currents recorded from CA1 pyramidal neurons in slices incubated in minocycline 500 nM for 1 h. CXCL16 was applied for 20 min. (**B**) Cumulative distribution curves showing no effect of CXCL16 (grey line) on both mIPSCs amplitude and IEI after treatment with minocycline. Inset: means of amplitude and frequencies (N = 6, Student’s t test, p > 0.05). (**C**) Representative recordings of mEPSCs in the same condition as in (**A**). (**D**) Cumulative distribution functions for mEPSCs amplitude and IEI. CXCL16 application is ineffecting on miniature EPSCs in minocycline treated slices. Histograms for mEPSCs amplitude and frequency are in the inset (N = 6), CTRL: black, CXCL16: grey.

Microglia is known to modulate synaptic transmission^40^,^41^,^42^, and microglia also express CXCR6^31^. We asked whether the changes in synaptic transmission observed upon CXCL16 administration might also required microglia neuron interplay. To test this hypothesis, slices were pre-treated for 1 h with the antibiotic minocycline (500 nM) to “inhibit” microglia activation^43^, and then continuously superfused during the recording. In this experimental condition, CXCL16-mediated modulation of GABAergic transmission was completely prevented, with no effect of CXCL16 on either mIPSCs amplitude (Fig. 7A, CTRL: 14.03 ± 0.97 pA, CXCL16: 14.12 ± 0.83 pA, N = 6, p = 0.84) or frequency (Fig. 7B CTRL: 2.8 ± 0.88 Hz, CXCL16: 3.03 ± 0.28 Hz, p = 0.12, N = 6). Similarly, we observed that, in the presence of minocycline, CXCL16 did not alter mEPSCs amplitudes (CTRL: 11.32 ± 1.02 pA, CXCL16: 11.74 ± 0.96 pA, p = 0.35, N = 6 Fig. 7D, left panel) or frequencies (CTRL: 0.42 ± 0.06 pA, CXCL16: 0.41 ± 0.09 pA, p = 0.89, N = 6, Fig. 7D, right panel). These experiments indicate that microglia play an important role in the hippocampal synaptic modulation mediated by the chemokine CXCL16.

## Discussion

CXCL16 is up-regulated in the brain upon different pathological states. Nevertheless, the observation that CXCL16 and its unique receptor CXCR6 are constitutively expressed in the CNS by neurons and glia suggests that this pair might have some important functions in brain homeostasis. In the present paper we describe for the first time that CXCL16 has neuromodulatory action in hippocampal CA1 region, tuning both the GABAergic and the glutamatergic transmissions, and that this modulation requires glia-neuron interplay.

At inhibitory synapses, CXCL16 enhances the action-potential independent release of GABA, increasing the frequency of mIPSCs. By contrast, the evoked release is reduced and the PPR ratio was increased. Interestingly, in the frequency distribution histogram of sIPSCs recordings, we found that, although CXCL16 did not affect the mean frequency, the number of small-sIPSCs (up to 20 pA, that resemble mIPSCs) was increased, whereas the largest were reduced, supporting the idea that CXCL16 increases the spontaneous GABA release while reduces the action-potential dependent ones. The opposite modulation of spontaneous and evoked release of neurotransmitter by different mediators has been previously reported^44^,^45^,^46^,^47^,^48^,^49^,^50^. Several mechanisms could contribute to the opposite regulation of spontaneous and evoked release. For instance, large differences in the amount of intraterminal Ca2 + levels have been shown to be associated with a block in evoked release or an increase in mEPSC frequency^51^,^52^. Moreover, differences in presynaptic fusion machineries giving rise to spontaneous and evoked release or segregation of spontaneous and evoked neurotransmission can account for the differential effects of CXCL16 on spontaneous and evoked GABA transmission^53^,^54^.

Alternatively, the enhancement of GABA release, associated with the increase in the frequency of mIPSCs, could activate presynaptic GABA-B receptors, reducing the probability of evoked release, as reported for hippocampal inhibitory neurons in CA3^55^. In line with this last hypothesis, we demonstrated that the pharmacological inhibition of GABA-B receptors prevents the effect of CXCL16 on evoked GABA release, suggesting their involvement in the mechanism underlying the opposite modulation by CXCL16 on spontaneous and evoked GABAergic activity.

Besides its role on GABAergic transmission, CXCL16 affects also excitatory transmission enhancing both spontaneous and evoked glutamate release. Specifically, we observed that CXCL16 increased the frequency of mEPSCs and the amplitude of eEPSCs but reduced the PPR, strongly indicating that CXCL16 enhances glutamate release.

**Figure 8:**
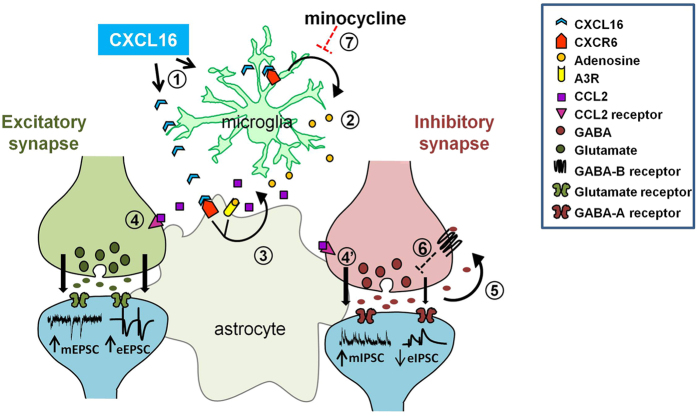
Schematic diagram summarizing the proposed mechanism of CXCL16 action. Exogenous CXCL16 might act on both microglia and astrocytic CXCR6 (1). Activation of microglial CXCR6 induces the release of adenosine (2). On astrocytes, the simultaneous activation of CXCR6, by exogenous CXCL16, and A3R, by adenosine, induces the release of CCL2 (3) that, acting presynaptically potentiates the spontaneous release (mEPSC) and evoked release (eEPSC) of glutamate on excitatory synapses (4). A similar mechanism might be involved in CXCL16 enhancement of spontaneous GABA release (mIPSC) at GABAergic synapses (4′). Increased GABA release activates presynaptic GABA-B receptors (5) reducing the probability of evoked release (eIPSC) (6). The inhibition of microglia activation with minocycline prevents the effects of CXCL16 on both glutamatergic and GABAergic transmission (7).

Presently, we are not able to distinguish whether CXCL16 acts directly on (i) glutamatergic and GABAergic terminals, (ii) via intermediate inhibitory interneuron(s), or (iii) by the action of glial cells, given the diffuse distribution of CXCR6^31^. However, the neuroprotective activity of CXCL16, which requires complex interplay between cell types^31^, suggest that glia-neuron interaction could be a general mechanisms of CXCL16 action. Supporting this complex interaction, we showed that inhibiting microglia function with minocycline, the effects of CXCL16 on GABAergic and glutamatergic transmission were abolished. It can be argued that minocycline can have additional effects on neurons^43^,^56^,^57^,^58^,^59^. However at the concentration we used (500 nM), minocycline did not alter basal synaptic transmission, because we did not observe differences in mIPSC and mEPSC amplitude and frequency. In addition we demonstrated that A3Rs are required for the neuromodulatory activity of CXCL16, since upon pharmacological or genetic inactivation of A3Rs, the chemokine is ineffective in modulating GABAergic and glutamatergic transmission. The relevance of A3Rs in mediating CXCL16 functions has been previously reported for the neuroprotective activity in ischemia^32^. Although the A3Rs are expressed in the brain, their function in the CNS is poorly understood^60^,^61^,^62^,^63^,^64^,^65^. Considering the low affinity of rat A3R for adenosine (Ki = 1 μM, ref. 66) it is generally thought that activation of A3Rs in the CNS might occur only under pathological conditions (such as hypoxia, ischemia or epilepsy) which increase extracellular levels of adenosine in the brain^65^,^67^,^68^,^69^. However, it has been recently shown that A3Rs can be tonically active in mouse hippocampal slices, tuning glutamatergic activity in combination with A1Rs and A2ARs^70^ but the consequences of A3Rs activation on basal synaptic transmission are poorly characterized. In the present paper, we propose A3R as key mediator of the presynaptic regulation of both GABA and glutamate release by CXCL16. Considering the widespread distribution of A3Rs, at present it is not clear whether neuronal or glial A3Rs are involved. Since it has been shown that A3R agonists potentiate excitatory post-synaptic currents both in cortical and hippocampal neurons, via post-synaptic mechanisms^7^,^64^,^70^,^71^, according to our data we can probably exclude the involvement of neuronal A3Rs in CXCL16-dependent modulation of glutamatergic transmission. In addition, as far as we know, there is only one paper that shows a link between A3Rs activation and the regulation of GABA release^72^, in an experimental model of pain, but the location of A3Rs has not been determined. We have previously demonstrated that, acting in concert with astrocytic A3R, CXCL16 mediates the release of CCL2 from astrocytes^31^. In the last years several evidences led the emerging concept that CCL2 may function as a neuromodulator in the CNS^73^,^74^, and in particular it has been shown that in the CA1 region of rat hippocampus CCL2 enhances neuronal excitability and synaptic transmission via presynaptic mechanisms^75^ with effects similar to those we found for CXCL16. According to all these findings we could speculate that the release of CCL2 from astrocytes could contribute to the modulation of glutamatergic and GABAergic neurotransmission by CXCL16 with a mechanism described in Fig. 8.

The increased excitation/inhibition ratio induced by CXCL16 is in apparent contrast with its neuroprotective activity^31^. However the two effects are induced by different CXCL16 concentrations and stimulation times, likely reflecting distinct mechanisms, not necessary correlated.

Overall, we demonstrated for the first time that CXCL16 is able to modulate neurotransmitter release on inhibitory and excitatory synapses in the hippocampus, with mechanisms requiring microglia and A3Rs activation. These data further reinforce the current idea that chemokines, key players in the interplay between neurons and glia cells, play unexplored roles in physiological conditions as regulators of synaptic transmission, representing endogenous modulators of brain homeostasis.

## Materials and Methods

### Animals

Procedures using laboratory animals were in accordance with the Italian and European guidelines and were approved by the Italian Ministry of Health in accordance with the guidelines on the ethical use of animals from the European Community Council Directive of 22 September 2010 (2010/63/EU). All efforts were made to minimize the number of animals used and their suffering.

Hippocampal slices were routinely obtained from 1-month-old C57BL/6 J. When specified, hippocampal slices were obtained also from: i) A3R knockout mice (A3RKO)^76^; ii) cxcr6 gfp knock-in mice^77^ in which the coding region of the receptor has been substituted with the coding region of the Green Fluorescent Protein (GFP). In the paper we refer to these mice as CXCR6KO mice: in particular heterozygous CXCR6KO were mated to obtain both homozygous CXCR6KO, and their CXCR6+/+ littermates as controls. Both A3RKO and cxcr6gfp knock-in mice were backcrossed at least 10 times on a C57BL/6J background.

Briefly, animals were decapitated after being anesthetized with halothane. Whole brains were rapidly removed from the skull and immersed in ice-cold gassed (95% O2, 5% CO2) sucrose-based artificial cerebrospinal fluid (ACSF) containing (in mM) 87 NaCl, 75 Sucrose, 2 KCl, 7 MgCl2, 0.5 CaCl2, 25 NaHCO3, 1.2 NaH2PO4 and 10 glucose, pH 7.4, 300–305 mOsm, then cut (ThermoScientific HM 650 V) in transversal slices (300 μm). Slices were placed in a slice incubation chamber at room temperature with oxygenated ACSF containing (in mM) 125 NaCl, 2 KCl, 1.2 MgCl2, 2 CaCl2, 25 NaHCO3, 1.2 NaH2PO4 and 10 glucose, pH 7.4, 300–305 mOms. After their preparation, slices were allowed to recover at least for 1 h before recording, then transferred to a recording chamber within 1–6 h after slice preparation.

### Patch clamp technique

Whole-cell patch clamp recordings were performed on CA1 pyramidal neurons at room temperature by using a Multiclamp 700B amplifier (Molecular Devices, USA). The ACSF was perfused at a rate of approximately 2 ml/min by using a gravity-driven perfusion system. Cell capacitance was constantly monitored over the time and experiments were access resistance changed more than 20% were discarded. Glass electrodes (3–4 MΩ) were pulled with a vertical puller (PC-10, Narishige). Pipette were filled with 140 mM Cs Methanesulfonate, 10 mM Hepes, 0.5 mM EGTA, and 2 mM Mg-ATP, Na3-GTP 0.3 mM, MgCl2 2 mM (295–300 mOsm, pH 7.2). Signals were acquired (sampling 10 kHz, low-pass filtered 2 kHz) with DigiData-1440A using pCLAMP-v10 software (Molecular Devices, USA).

GABAergic outward membrane currents were recorded with the neuron clamped at 0 mV. At this voltage, Cl−-mediated inhibitory events are outward currents (estimated ECl = −80 mV) whereas excitatory currents are inward but of small amplitude as they would occur close to their reversal potential. Although it was possible to isolate sIPSCs pharmacologically, by using 10 μM CNQX plus 50 μM AP-5 to block both the AMPA and NMDA receptor components of spontaneous excitatory postsynaptic currents (sEPSCs), this antagonist mixture sometimes attenuated or occasionally completely blocked sIPSCs^78^. This presumably reflected impediment of excitatory synaptic drive to the inhibitory interneurons that were responsible for sIPSC generation. In view of this variable effect of CNQX/AP-5 on sIPSCs, we elected to use a holding potential of 0 mV rather than pharmacological methods to separate sIPSCs from sEPSCs. The validity of this approach is supported by the observation that 100μM picrotoxin completely eliminated all spontaneous outward current activity recorded at 0 mV (not shown).

By using the same conditions, excitatory post-synaptic currents (EPSCs) were recorded clamping the cell at −80 mV. In a subset of experiments, the glutamatergic nature of the mEPSC recordings was confirmed at the end of the experiment by total blockade of mEPSCs by DNQX (20 μM; data not shown).

### Miniature recordings

Miniature EPSCs/IPSCs were recorded during an initial 10 min baseline period, followed by application of TTX (0.5μM, Tocris Bioscience, Bristol, United Kingdom) for 15 min. After stabilization of TTX effect, CXCL16 was applied for 20 min. Only data from the last 5 min of each recording epoch was analyzed to ensure that drugs had fully equilibrated. Analysis was performed off-line using MiniAnalysis software (Mini Analysis, Synaptosoft Fort Lee, NJ, USA) with the threshold for detection set at 5 pA. Whole-cell currents were recorded at 20 kHz and filtered with a Bessel filter at 4 kHz.

### Paired-pulse experiments

A concentric bipolar stimulating electrode (SNE-100 × 50 mm long Elektronik-Harvard Apparatus GmbH, Crisel Instruments, Rome, Italy) was positioned in the stratum radiatum to evoke IPCSs from CA1 pyramidal neurons or in CA3 region to evoke eEPSCs. Pairs of stimuli (ISI 50 ms) were applied every 20 sec. Stimulus intensity was 0.27 ± 0.03 mA (range, 0.2–0.4 mA), delivered through a A320R Isostim Stimulator/Isolator (WPI). PPR was calculated as the ratio between the amplitude evoked by the second stimulus (A2) over the first (A1; A2/A1) and the amplitude of each EPSC/IPSC was measured relative to a 2 ms long baseline period starting 3 ms before stimulation.

### Drugs

CXCL16 (murine, Peprotech Inc. Rocky Hill, NJ, USA), Tetrodotoxin citrate (Tocris, Bristol, UK, stock solution 1 mM in ethanol), CGP 55845 hydrochloride (Tocris, Bristol, UK, stock solution 10 mM in DMSO), MRS 1523 (Sigma Aldrich, Saint Louis, Missouri, USA, stock solution 10 mM in DMSO). Ultrapure water was prepared using a Milli-Q system (Millipore, MA, USA). Drugs were dissolved in ACSF just before application.

### Statistical analysis

The values were reported as mean ± SEM. Unless otherwise specified, data values refer to number of cells analyzed. Student’s paired t tests was used for inter-group comparison. For cumulative probability plot comparisons we used Kolmogorov-Smirnov test (Mini Analysis, Synaptosoft Fort Lee, NJ, USA). Levels of significance were set as *p < 0.05; **p < 0.01; ***p < 0.001.

## Additional Information

**How to cite this article**: Di Castro, M.A. *et al*. The chemokine CXCL16 modulates neurotransmitter release in hippocampal CA1 area. *Sci. Rep*. **6**, 34633; doi: 10.1038/srep34633 (2016).
